# Using neural networks to predict the effect of the preload location on the natural frequencies of a cantilever beam

**DOI:** 10.1016/j.heliyon.2022.e11242

**Published:** 2022-10-29

**Authors:** Ahmed M. Paridie, Nicoleta M. Ene, Yasser S. Mohamed

**Affiliations:** a University of Toledo, College of Engineering, MIME Department, USA; b Alexandria University, Faculty of Engineering, Department of Mechanical Engineering, Egypt

**Keywords:** Optimization, FEM, Artificial intelligence, Modal analysis, Neural networks, Beams

## Abstract

With the evolution of computational power of computers in 20th century, neural networks (NNs) are becoming more popular in different engineering applications because of its ability to approximate static and dynamic, linear and non-linear, multi-dimensional systems. For example, they are used in industrial processes to automate assembly lines which increases its productivity and in automotive to reduce gas consumption of an engine. In this paper, NNs are utilized to reduce the computational power needed for finite element methods (FEM) simulations. A case study is taken for which NNs are used to predict the effect of the preload position and magnitude on the natural frequencies of the prestressed cantilever beam. A simple FEM model is implemented to generate the data set required to train the NN. The steps done to construct the FEM are discussed and the FEM model results are verified. The effect of the preload position on the natural frequencies of the beam is studied. A NN is then implemented to predict the natural frequencies of the beam for different beam cross-section geometries and different preload magnitudes and positions. The NN architecture, data processing and training methodology are explained. The NN and FEM results are compared to show the accuracy of the NN predictions. The results are shown to be in good agreement.

## 1 Introduction

Predicting a problem output for all possible combinations of inputs within a given inputs domain using finite element method (FEM) consumes time and computational power (Silva et al., 2021; Alefe et al., 2020). To minimize the time and power consumed by FEM, Neural networks (NNs) are proposed as function approximators to predict the problem output for any input combination (Bohn et al., 2013; Javadi et al., 2009). This approach is proven to be a time and power consumption efficient solution (Qi et al., 2019; Mou et al., 2020). To emphasize the benefits of using NN to predict FEM results, a generalization of the work done by Ozturk (2011) and Ang et al. (1993) is performed in this paper. Ozturk (2011) measured the effect of the magnitude of a tip preload on the natural frequencies of a prestressed cantilever beam. In this paper, a contribution is done in this paper by studying the effect of the preload magnitude and position on the natural frequencies of the prestressed cantilever beam as shown in Figure 1-1. An advantage of changing the position of the load application point in addition to the load magnitude adds an additional degree of freedom is obtaining a wider range of natural frequencies that can be obtained for the same force magnitude.

**Figure 1-1:**
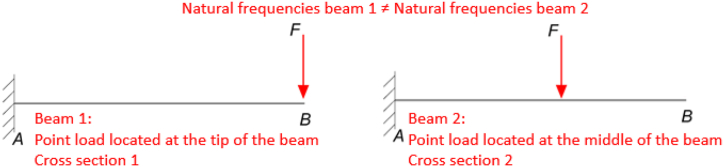
Different beam configurations that has different natural frequencies.

A simple FEM model to study the effect of the preload position on the natural frequencies of the beam and to generate a data set to train the NN (Kononenko et al., 2018) is constructed, explained, and verified. The dataset generated by the verified FEM model is then used to construct a model that predicts output for any input combination that falls within a given inputs domain (Capuano and Rimoli, 2019; Gulikers, 2018). For example, if the NN is trained to predict the natural frequencies for a beam stiffness ranging from 0.1 inch to 10 inches, the input beam stiffness to the NN should be within this range. Using this approach, the number of simulations done by FEM to find all possible combinations is dramatically reduced (Arnd, 2021; German, 2019). For example, if a problem has 4 inputs with 15 discrete values per each input, the number of simulations required to predict all possible outputs is 15^4^ or 3375 simulations. When using a NN in this paper, only 198 simulations are done, and the results of these simulations are used to train a NN to predict the outputs for all possible combination of inputs that fall within the given inputs domain as demonstrated in Figure 1-2.

**Figure 1-2:**
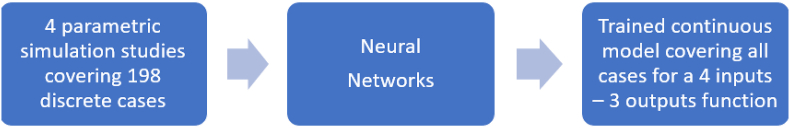
Neural networks advantage demonstration.

## 2 A simple FEM model to study the effect of the preload position on the natural frequencies of a cantilever beam

Before studying the effect of the preload position on the natural frequencies of the prestressed cantilever beam, the results obtained by Ozturk (2011) are verified using a simpler FEM model (Capuano and Rimoli, 2019). The advantage of this simpler FEM model is the ability to obtain quicker results for different preload positions and magnitudes. The same material properties used by Ozturk (2011) are adopted. Instead of implementing two studies as done by Ozturk (2011) to obtain the beam deformed shape then the deformed beam natural frequencies, in this paper, the beam is implemented as an elastic curve described by Figure 2-1 mentioned in (NCEES, 2020, page 141) which represent the deflection of Euler cantilever beam under concentrated load. To model the concentrated preload, a fixed constraint is implemented at the point of the application of the preload, then, an eigen frequency solver is used to obtain the first three natural frequencies of the prestressed cantilever beam.

**Figure 2-1:**
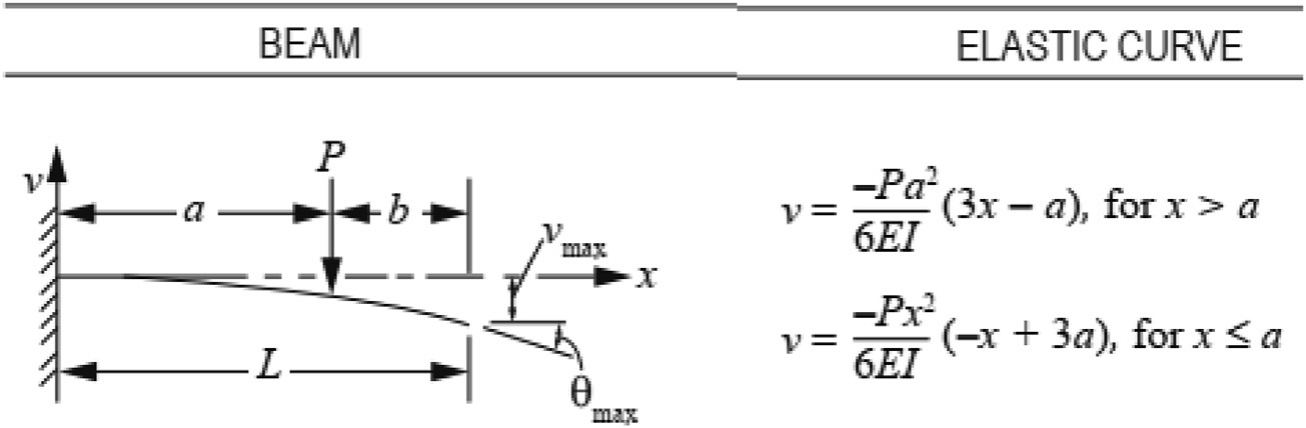
Cantilever beam elastic curve.

The inputs domain of this model is a beam width (b) ranging from 15 mm to 45 mm, beam thickness (t) ranging from 1 to 3 mm, load parameter (β) ranging from 0 to 1 implemented in Eq. (1) (Ozturk, 2011) by and loading position (x) ranging from the fixed end to the free end of the cantilever beam of 0.75 m length.

Eq. (1) $$P=\frac{\beta EI}{L^{3}}$$

where

P vertical load at position (x)
B the load parameter
E modulus of elasticity
I moment of inertia of the ross-sectional area of the beam bout the axis of bending
L length of the beam

After constructing the FEM model, a frequency domain-parametric study is done to obtain the data required to train the NN and to verify for the results of the simpler FEM model (Liang et al., 2018). The FEM study is done using commercial software for all combinations of the following discrete values:

• Beam thicknesses of 1, 2 and 3 mm
• Beam width of 15–45 mm with a step of 3 mm
• Loading factor (β) of 0–0.5 with a step of 0.1

Figures 2-2, 2-3 and 2-4 shows that the first three natural frequency parameters (λ_1_, λ_2_, λ_3_) expressed by Eq. (2) (Ozturk, 2011) are in good agreement with Ozturk (2011) results.

Eq. (2) $$\lambda_{i}=\omega_{i}\sqrt{\frac{\rho AL^{4}}{EL}}$$

β Load parameter
A Beam cross sectional area

**Figure 2-2:**
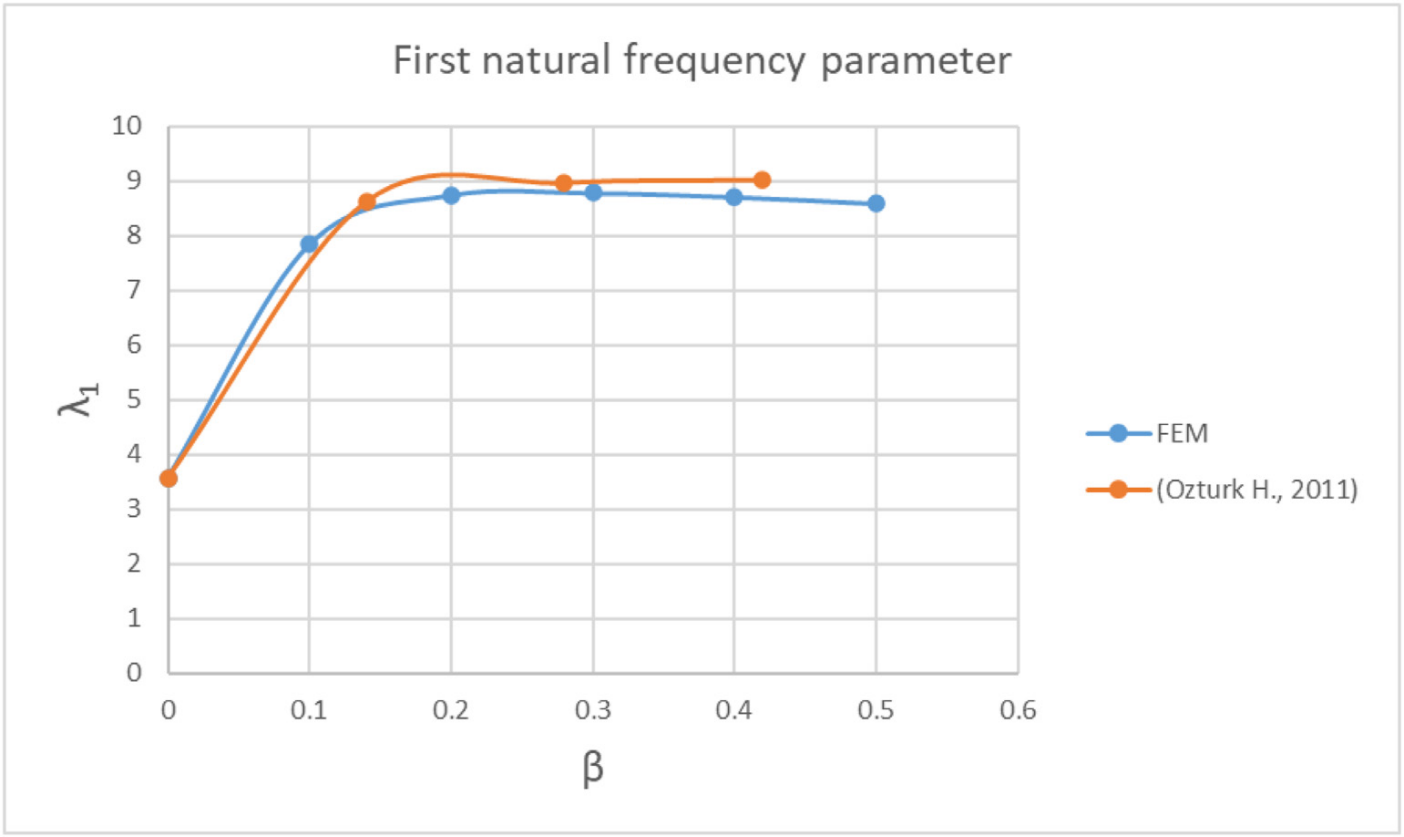
First natural frequency parameter FEM vs Ozturk (2011) results.

**Figure 2-3:**
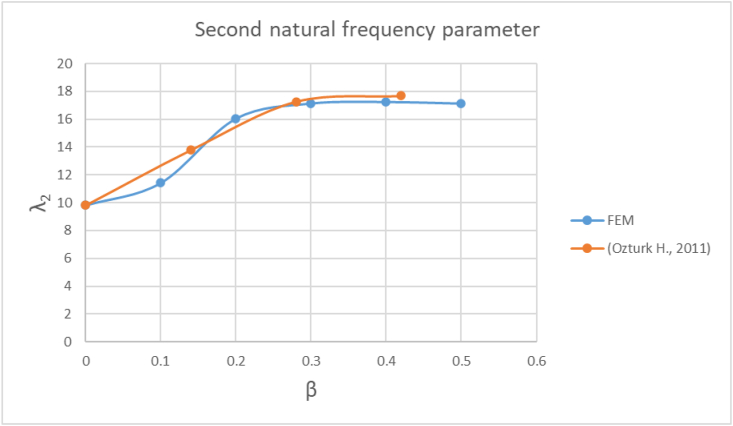
Second natural frequency parameter - FEM vs Ozturk (2011) results.

**Figure 2-4:**
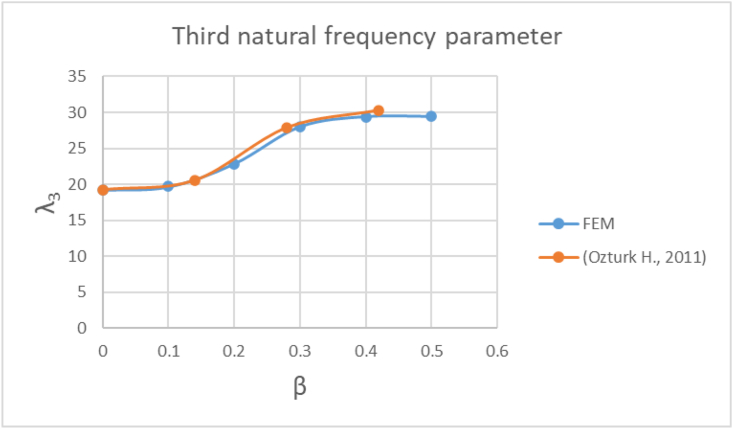
Third natural frequency parameter FEM vs Ozturk (2011) results.

Since this FEM model is verified, it can be used to study the effect of the effect of the preload position on the natural frequencies of the prestressed cantilever beam for the same preload magnitude and to train the NN to predict the natural frequencies for any combination of cantilever beam cross section width and thickness and for any preload magnitude and position that fall within the problem inputs domain.

**Figure 3-1:**
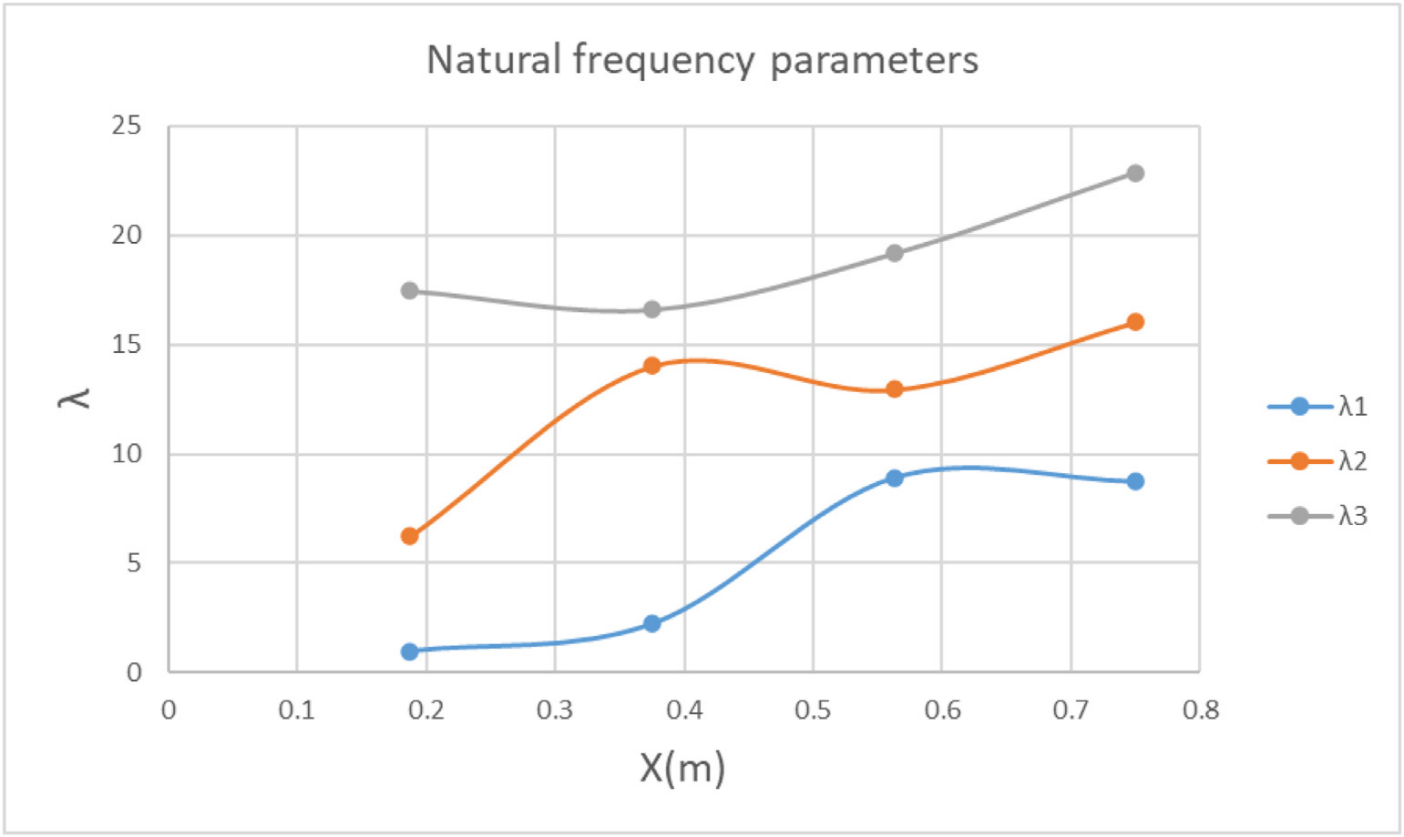
λ at a beam of 2 mm thickness, 30 mm width, preload factor of 1 applied and point load positioned at different locations on the beam.

From the FEM results at different preload positions (x) and the same preload magnitude shown in Figure 3-1, it is noted that for the same beam cross section and preload magnitude, the natural frequency tends to decrease as the preload position gets closer to the cantilever beam fixed end. The decreasing pattern is noted to be nonlinear, therefore, NN come in action as function approximators (Qi et al., 2019) to predict the effect of the preload location and magnitude on the natural frequencies of the cantilever beam.

## 3 Neural network predictions

To predict first three natural frequencies of the prestressed cantilever beam for all possible combinations of inputs within given inputs domain, a multilayer perceptron neural network (MLP) with a “relu” activation function is implemented using tensor flow library on python to perform non-linear regression (Javadi et al., 2009). The reason an MLP NN is chosen is because of the simplicity of the training algorithm used to train the NN compared to advanced NNs. Other NNs could have been used but the NN training time would be longer to obtain similar results (Lee et al., 2020). The NN inputs are the geometrical parameters of the cross section of the beam and the vertical concentrated load magnitude and position. The NN network output is the preloaded beam first three natural frequencies. To prepare the simulation data obtained from the FEM simulations for the NN training process, the FEM data are pre-processed (Mou et al., 2020). The steps to pre-process the dataset are shuffling, normalizing, and splitting the dataset into training, validation, and test datasets. The training data and validation are used to train the NN while the test dataset is used to indicate the ability of the NN to describe the physics of the physical system by measuring the mean square error (MSE) of the NN (German, 2019). After training the NN, the NN results are denormalized and the MSE is measured for the test dataset (Lee et al., 2020).

Figures 4-1, 4-2 and 4-3 displays FEM results vs NN outputs for natural frequency parameters at different beam cross section geometrical parameters, loading magnitude and position. Since there are four different inputs, a 2D plot is generated by plotting the loading factor β on the horizontal axis. The trained NN is able to predict the physics of the FEM model.

**Figure 4-1:**
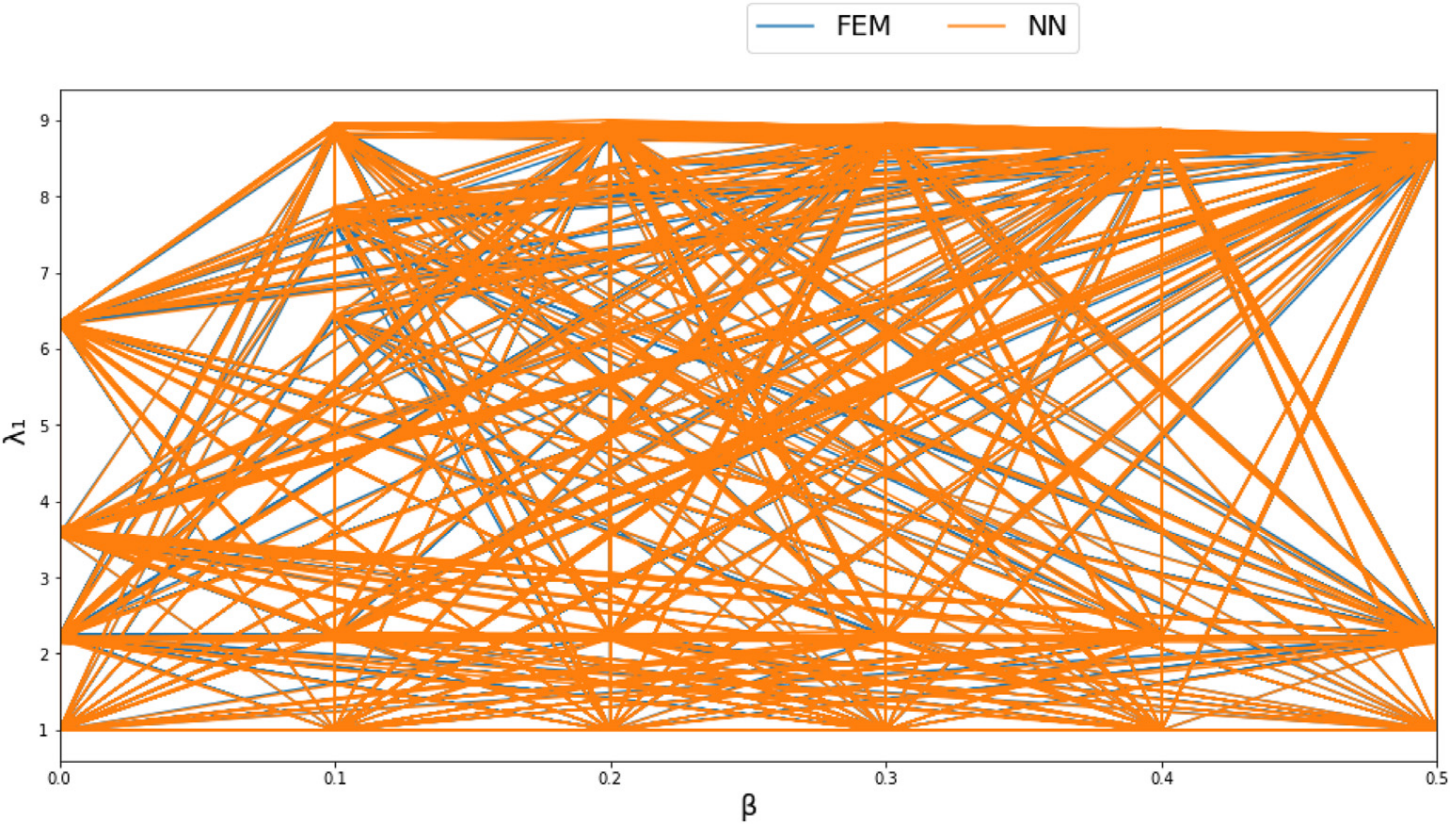
λ_1_ at different beam width and thickness and different loading magnitude and position.

**Figure 4-2:**
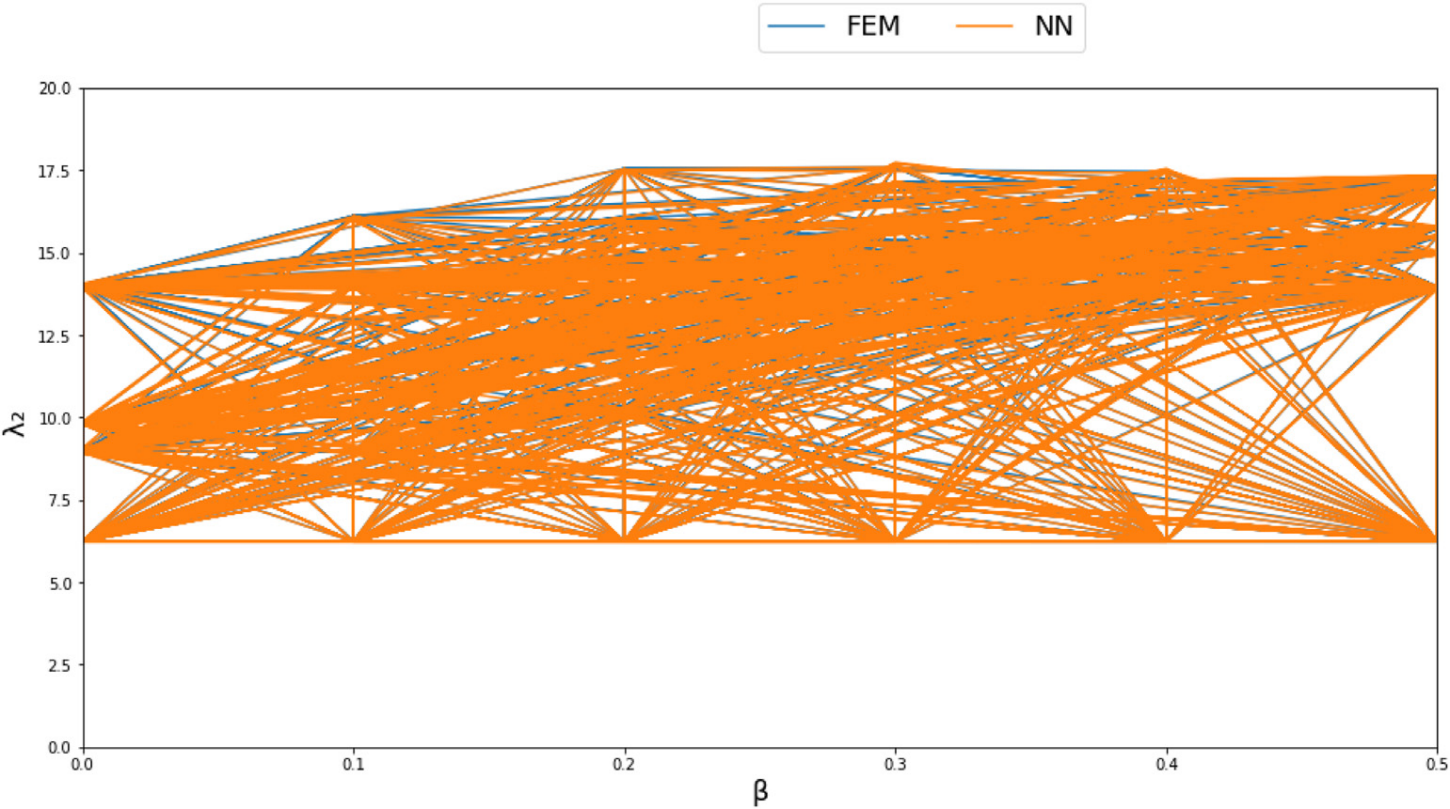
λ_2_ at different beam width and thickness and different loading magnitude and position.

**Figure 4-3:**
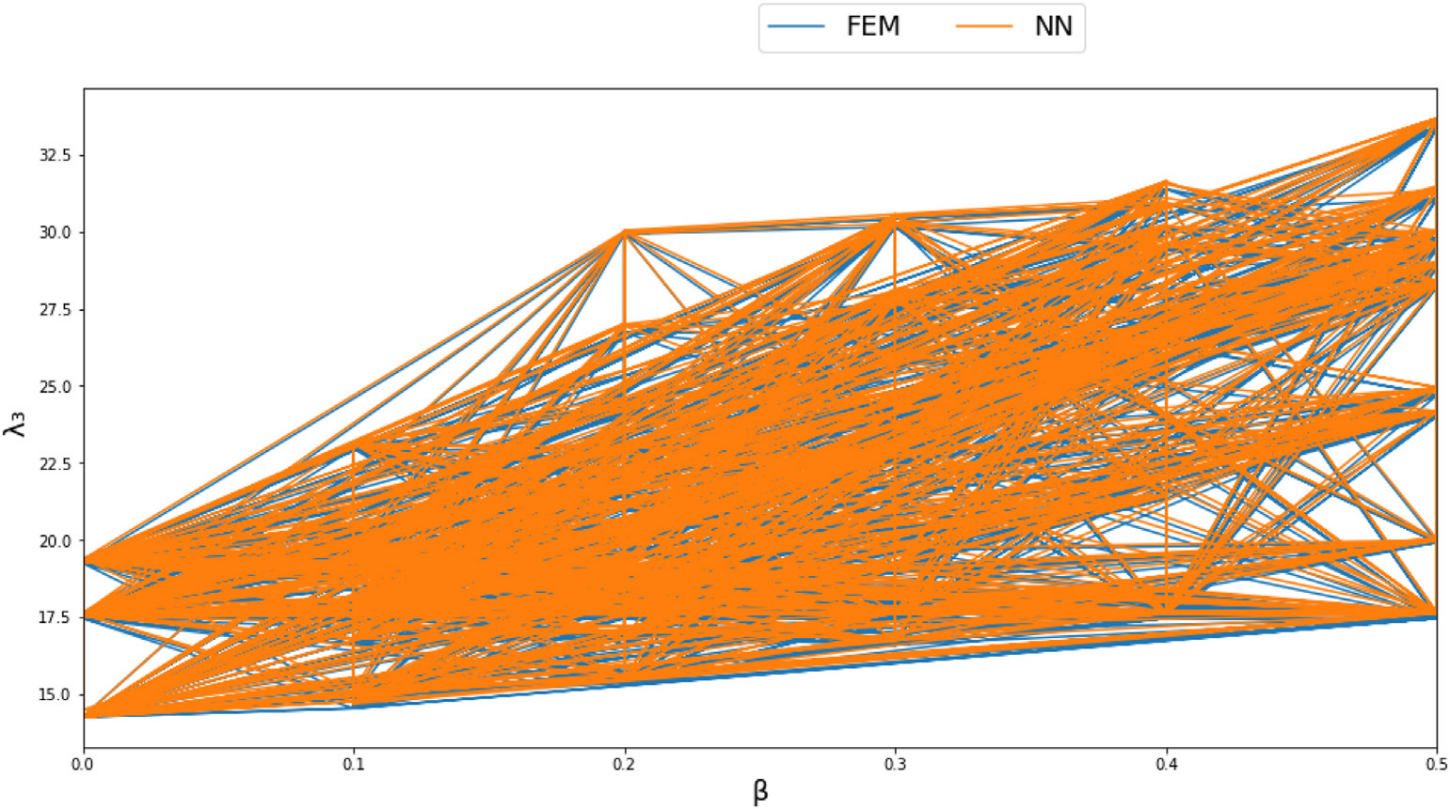
λ_3_ at different beam width and thickness and different loading magnitude and position.

To give a clearer demonstration on the performance of the NN, several input parameters are fixed to obtain a clear graph. The natural frequency parameters are plotted at a beam cross section of 15 mm width, 3 mm thickness and a point load located far from the fixed end by a distance equal to quarter the length of the beam as shown in Figures 4-4, 4-5 and 4-6.

**Figure 4-4:**
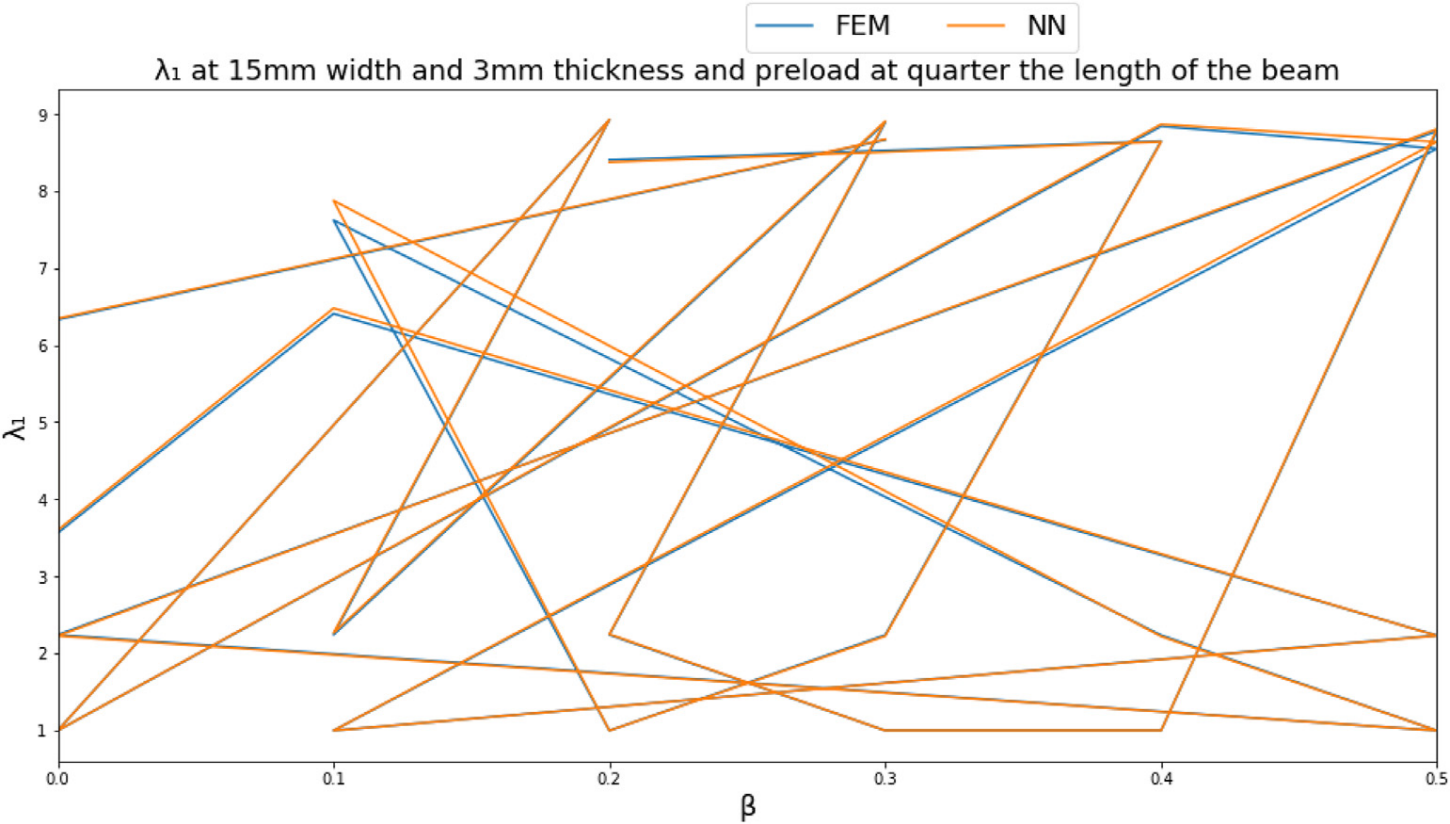
λ_1_ at a beam cross section of 15 mm width, 3 mm thickness and a point load located far from the fixed end by a distance equal to quarter the length of the beam.

**Figure 4-5:**
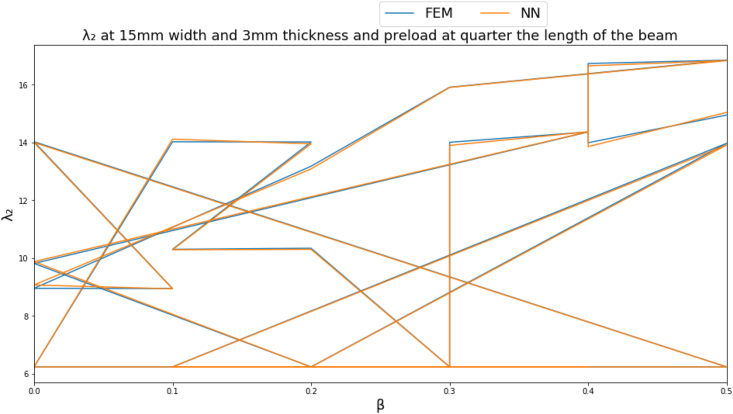
λ_2_ at a beam cross section of 15 mm width, 3 mm thickness and a point load located far from the fixed end by a distance equal to quarter the length of the beam.

**Figure 4-6:**
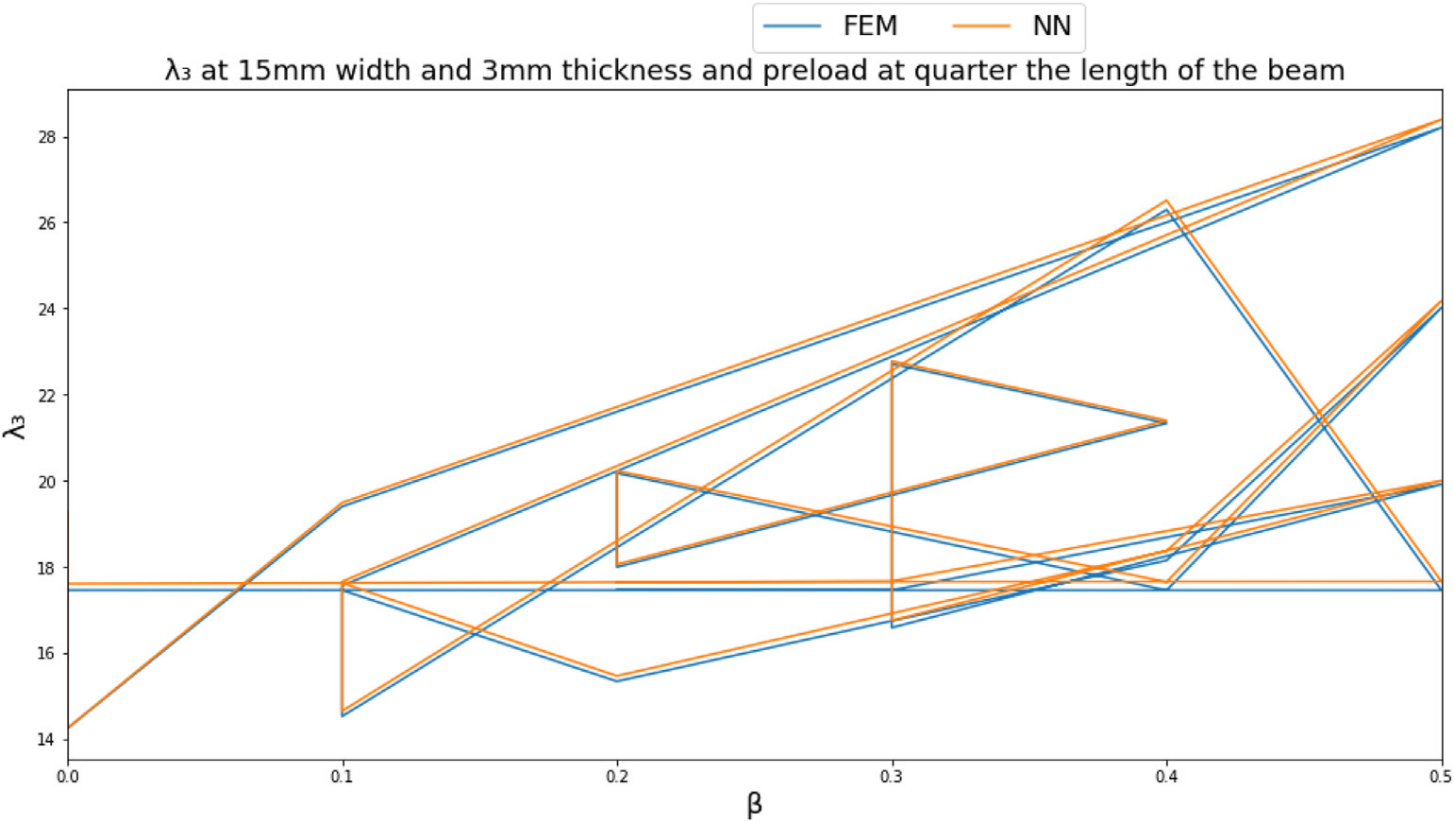
λ_3_ at a beam cross section of 15 mm width, 3 mm thickness and a point load located far from the fixed end by a distance equal to quarter the length of the beam.

As a final verification step for the trained NN model, NN results are plotted against FEM results in Figures 4-7, 4-8 and 4-9 for a beam cross section of 15 mm width, 3 mm thickness and a point load applied at the free end of the beam which are the same conditions adopted by Ozturk (2011).

**Figure 4-7:**
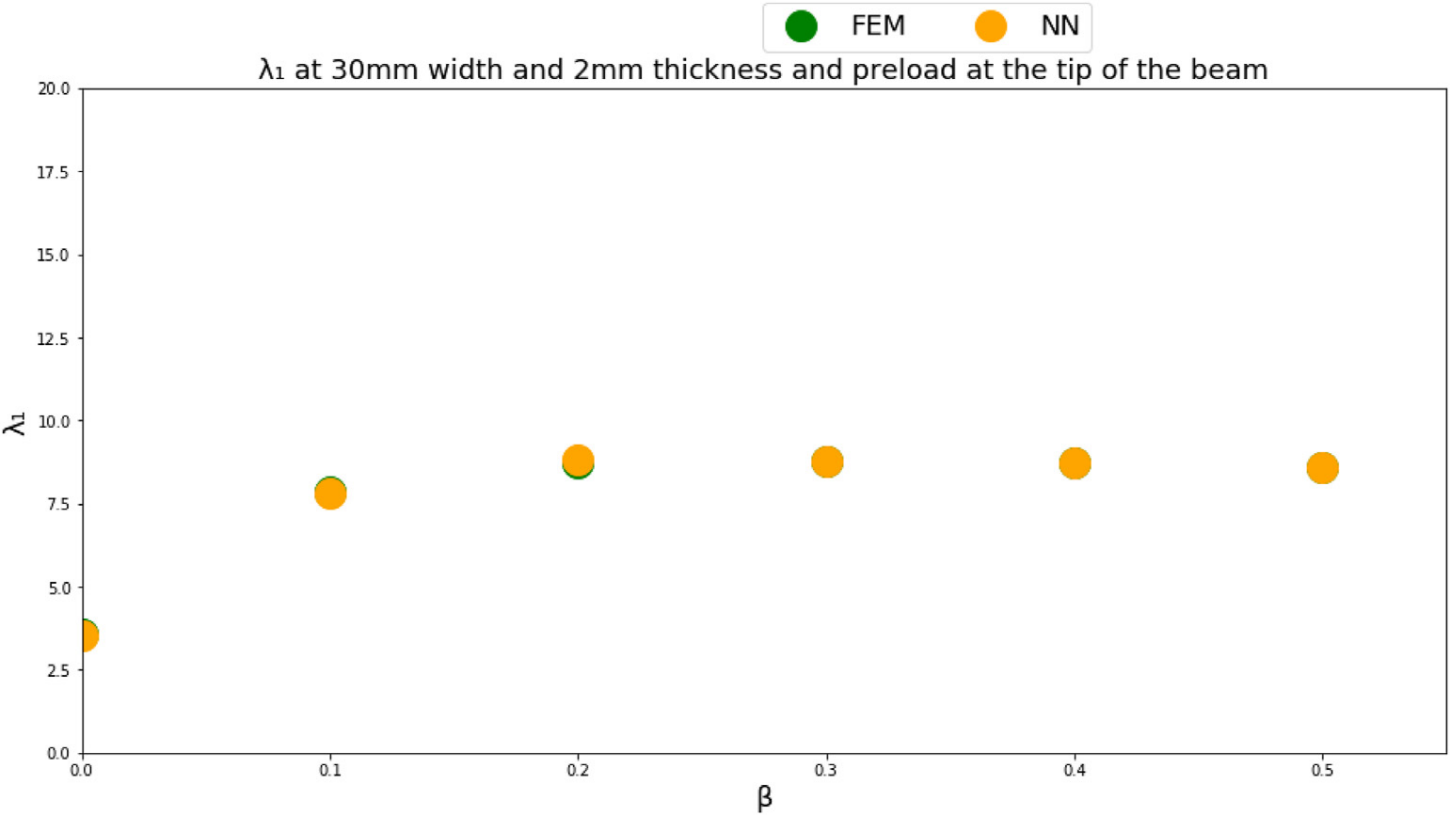
λ_1_ at a beam cross section of 15 mm width, 3 mm thickness and a point load applied at the tip of the beam.

**Figure 4-8:**
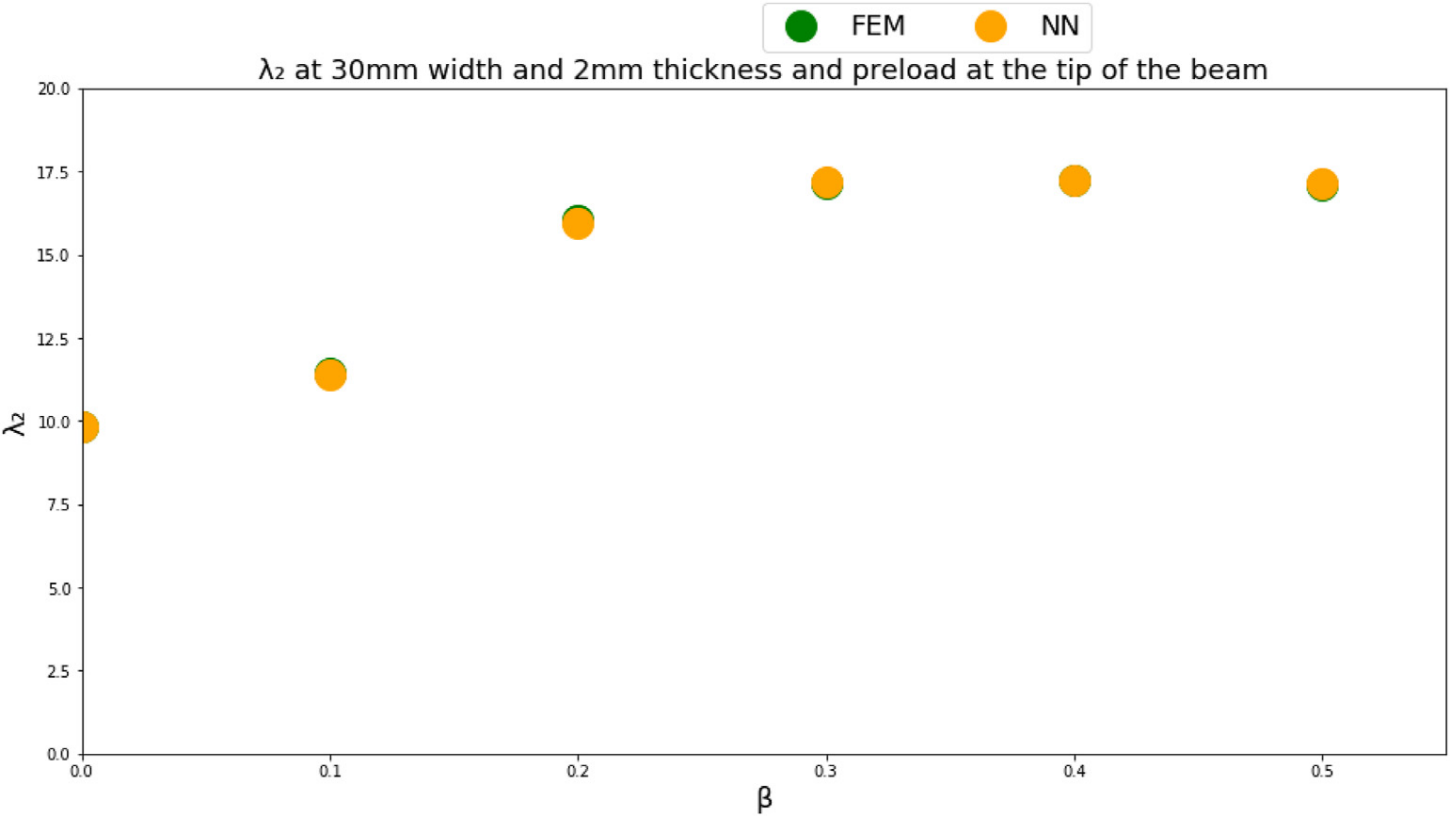
λ_2_ at a beam cross section of 15 mm width, 3 mm thickness and a point load applied at the tip of the beam.

**Figure 4-9:**
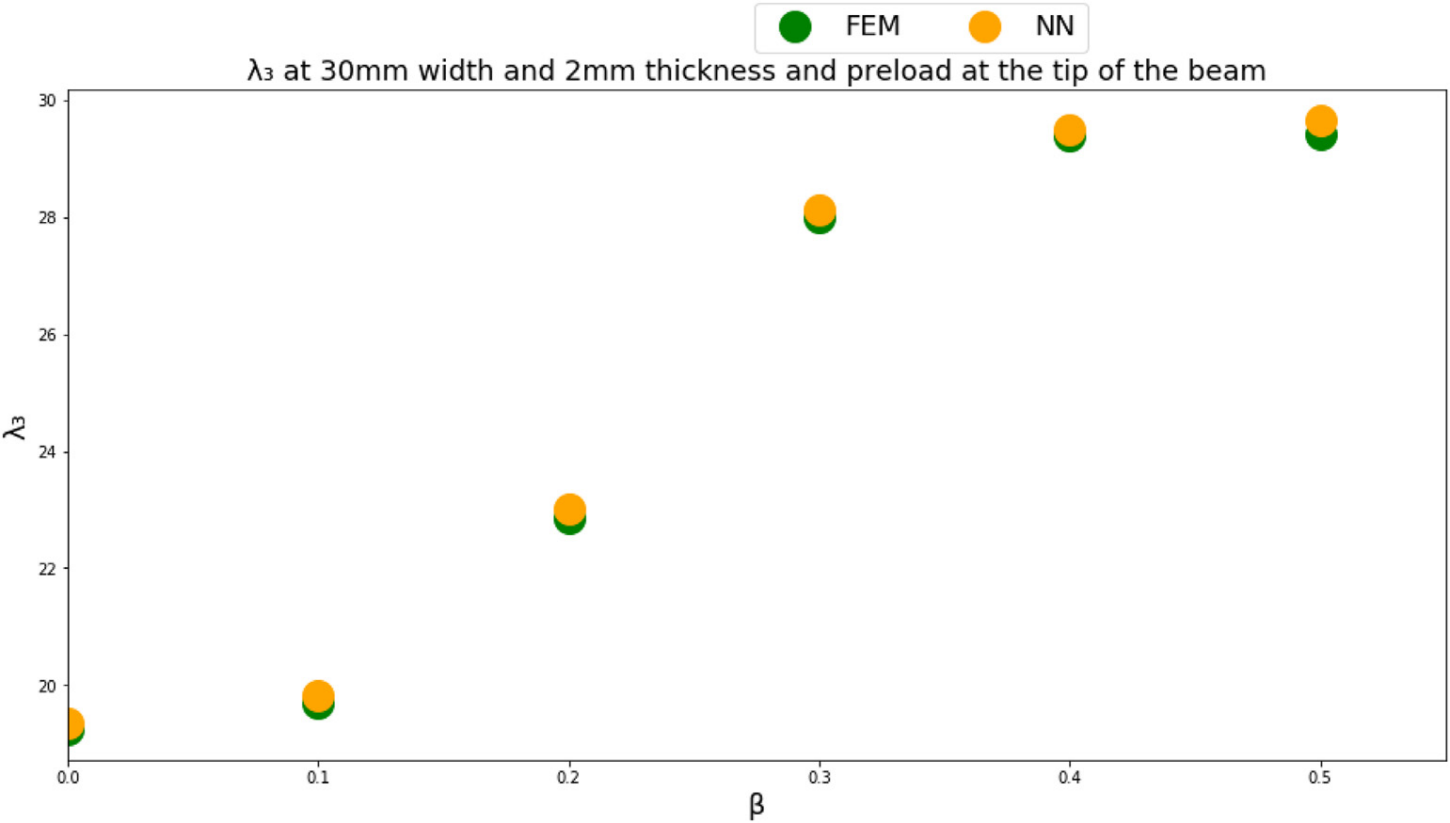
λ_3_ at a beam cross section of 15 mm width, 3 mm thickness and a point load applied at the tip of the beam.

The NN predictions are in good agreement with the FEM results where the MSE is less than 1% for all predicted natural frequencies which indicates that a multi-layer perceptron NN succeeded in approximating the physics of the problem for a given inputs domain. A limitation though of the NNs is that the NN inputs should fall within the given inputs domain used to train the NN.

## 4 Conclusion

A simpler FEM approach is implemented to predict the first three natural frequencies of the cantilever beam for different beam cross section geometrical parameters and different preload positions and magnitudes. The FEM model is verified by comparing its results to the results of Ozturk (2011) and the effect of the preload position on the natural frequencies of the cantilever beam is studied. It is concluded that the natural frequencies tend to non-linearly decrease as the point load gets closer to the fixed end of the cantilever beam. A NN is trained using the verified FEM model results to predict the first three natural frequencies of the cantilever beam for all possible combinations of the given input parameters is implemented and verified.

A further progress that can be done after this point is to use an inverse mapped NN to predict the beam geometry, preload position and magnitude that would result from a given input natural frequency. This way, the required geometry, preload position and magnitude combinations that need to be avoided to avoid a certain natural frequency can be predicted.

## Declarations

### Author contribution statement

Ahmed M. Paridie: Conceived and designed the experiments; Performed the experiments; Analyzed and interpreted the data; Contributed reagents, materials, analysis tools or data; Wrote the paper.

Nicoleta M. Ene; Yasser S. Mohamed: Contributed reagents, materials, analysis tools or data.

### Funding statement

This research did not receive any specific grant from funding agencies in the public, commercial, or not-for-profit sectors.

### Data availability statement

Data included in article/supp. material/referenced in article.

### Competing interest statement

The authors declare no conflict of interest.

### Additional information

No additional information is available for this paper.
